# Early prediction of spontaneous preterm birth before 34 gestational weeks based on a combination of inflammation-associated plasma proteins

**DOI:** 10.3389/fimmu.2024.1415016

**Published:** 2024-07-15

**Authors:** Maria Svenvik, Johanna Raffetseder, Lars Brudin, Göran Berg, Sandra Hellberg, Marie Blomberg, Maria C. Jenmalm, Jan Ernerudh

**Affiliations:** ^1^ Department of Obstetrics and Gynecology, Region Kalmar County, Kalmar, Sweden; ^2^ Department of Biomedical and Clinical Sciences, Linköping University, Linköping, Sweden; ^3^ Department of Clinical Physiology, Region Kalmar County, Kalmar, Sweden; ^4^ Department of Health, Medicine and Caring Sciences, Linköping University, Linköping, Sweden; ^5^ Department of Obstetrics and Gynecology, Linköping University, Linköping, Sweden; ^6^ Department of Physics, Chemistry and Biology, Linköping University, Linköping, Sweden; ^7^ Department of Clinical Immunology and Transfusion Medicine, Linköping University, Linköping, Sweden

**Keywords:** preterm birth, prediction model, inflammation, plasma proteins, proximity extension assay

## Abstract

**Background:**

In order to identify and possibly offer prophylactic treatment to women at risk for preterm birth (PTB), novel prediction models for PTB are needed. Our objective was to utilize high-sensitive plasma protein profiling to investigate whether early prediction of spontaneous PTB (sPTB) before 34 gestational weeks (gw) was possible in a low-risk population.

**Methods:**

A case-control study was conducted on 46 women with sPTB before 34 gw and 46 women with normal pregnancies and term deliveries. Prospectively collected plasma sampled at gw 11 (range 7-16) and gw 25 (range 23-30) was analyzed with a high-sensitivity Proximity Extension Assay for levels of 177 inflammation-associated proteins, and statistically processed with multivariate logistic regression analysis.

**Results:**

In the first trimester, higher levels of hepatocyte growth factor (HGF) were associated with sPTB <34 gw (OR 1.49 (1.03-2.15)). In the second trimester, higher levels of interleukin (IL)-10 (OR 2.15 (1.18-3.92)), IL-6 (OR 2.59 (1.34-4.99)), and the receptor activator of nuclear factor κB (RANK) (OR 2.18 (1.26-3.77)) were associated with sPTB <34 gw. The area under the curve for the prediction models including these proteins was 0.653 (0.534-0.759) in the first trimester and 0.854 (0.754-0.925) in the second trimester.

**Conclusion:**

A combination of inflammation-associated plasma proteins from the second trimester of pregnancy showed a good predictive ability regarding sPTB before 34 gw, suggesting it could be a valuable supplement for the assessment of the clinical risk of sPTB. However, although a high number (n=177) of plasma proteins were analyzed with a high-sensitivity method, the prediction of sPTB in the first trimester remains elusive.

## Introduction

Preterm birth (PTB), delivery before 37 gestational weeks (gw), is the leading cause of neonatal morbidity and mortality, and the most common cause of death for children under five years of age (1). The underlying mechanisms of PTB are still elusive, especially in the more than 50% of cases that are unexplained. Roughly, one-third of PTBs are medically indicated due to preeclampsia, fetal growth restriction, placental complications, or other reasons, whereas spontaneous PTB (sPTB) constitutes about two-thirds of the cases. There are different phenotypes leading to sPTB; either preterm labor (PTL), a condition with preterm uterine contractions and cervical ripening, or preterm prelabor rupture of the membranes (PPROM) (2–7). Generally, these two conditions are regarded as having different underlying mechanisms although inflammatory changes are considered important in both cases, either due to microbial-induced inflammation or immunological discontinuance of fetal-maternal tolerance (3).

The possibilities to identify women at high risk for sPTB in early pregnancy are limited. For instance, prediction models based on maternal characteristics were not able to adequately predict sPTB in a low-risk population (8). Cervical length screening in the second trimester by transvaginal ultrasound to predict PTB offers only moderate value in populations with a low prevalence of PTB (9). As there is evidence that prophylactic treatment with progesterone may be beneficial in certain situations (10–12), biomarkers to improve the predictability of sPTB are highly needed. A large genetic study recently revealed associations between several genetic variants and gestational length as well as PTB (13), offering improved mechanistic insights into the syndrome of PTB and opening possibilities to discover other prophylactic treatment options than progesterone. Furthermore, it has been shown that pregnancy is associated with fine-tuned and precisely timed changes in subsets of immune cells (14), in the serum metabolome (15), as well as in the plasma proteome (16, 17), indicating the possibility of identifying protein biomarkers in early pregnancy that might offer a prediction model for sPTB. It would be particularly important to distinguish the pregnancies at risk for sPTB before 34 gw, which is the period with the greatest risk of sequelae for the neonate and before which corticosteroids are usually given to decrease respiratory complications.

Previous work has suggested prediction models based on maternal characteristics in combination with biomarkers (18, 19), transcriptomics (20, 21), proteomics (20, 22, 23), metabolomics (15, 24), inflammatory lipid biomarkers (25), transcripts of cell-free RNA in maternal blood (26), as well as circulating microparticles (27). However, for the discovery of protein biomarkers, robust measurements at a high sensitivity are essential since several relevant immune proteins occur at low levels in the maternal circulation. These requirements are met by using the Proximity Extension Assay technique, which offers the possibility to reliably detect a broad array of plasma proteins with high sensitivity and specificity (28, 29) in a very small sample volume (one μL). Thus, the Proximity Extension Assay would be suitable for the identification of plasma proteins possibly associated with subsequent sPTB in a cohort of pregnant women.

The aim of this study was to evaluate whether prediction of sPTB prior to 34 gw was possible in a low-risk population of pregnant women, based on the levels of inflammation-associated plasma proteins in the first and second trimesters, measured by a high-sensitivity detection assay.

## Materials and methods

### Study population and collection of samples

During the years 2011 – 2018, blood samples were consecutively collected from pregnant women at two antenatal clinics in the county of Östergötland, Sweden. A biobank was created by collaborators at the Linköping University Hospital and Linköping University, Sweden, to enable research on pregnancy complications among a population of predominantly low-risk women. The pregnant women received information about the research project when booking their first appointment at the antenatal clinic and received further information at their first visit. The women who agreed to participate in the study gave written informed consent to donate blood samples in the first and second trimesters as well as at delivery, and to permit researchers to access their present and future medical records. The biobank was approved by the Regional Ethical Review Board in Linköping, Sweden (2010/296-31 and 2019-00424).

In total, 8,027 women agreed to contribute to the biobank. During this period the two antenatal clinics registered visits from 19,294 unique women in total, which constituted >95% of the pregnant women in this region of Sweden during the study period.

**Figure 1:**
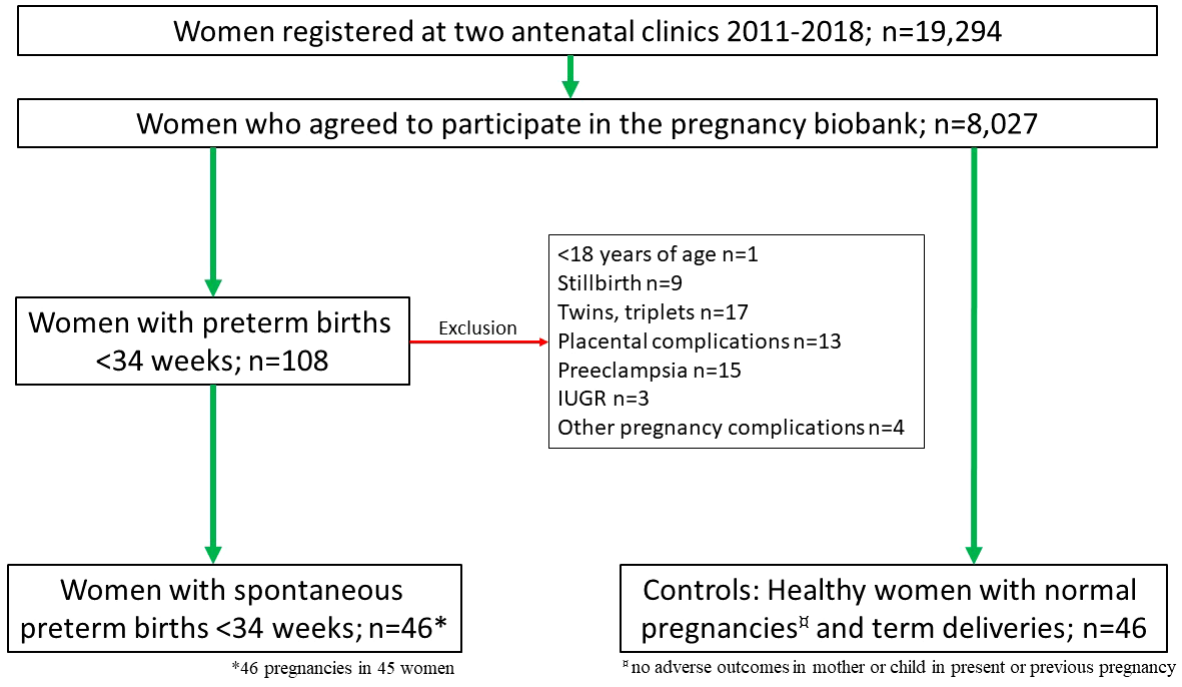
Flow chart of the women included in the study with exclusion criteria stated (red arrow).

A case-control study using the registered information and prospectively collected blood samples was performed. To identify women with PTB, all women in the biobank (n=8,027) with at least one PTB prior to 34 gw were identified (n=108) by linking the database of the biobank to the Obstetrix electronic medical records database (Cerner Sweden Corp.). One woman who was below 18 years of age was excluded. The medical records of the remaining 107 cases were scrutinized to identify women with sPTB before 34 gw. Exclusion criteria were stillbirth (n=9), twins (n=16) or triplets (n=1), placental complications (abruption (n=9) or placenta praevia (n=4)), and medically indicated PTB due to preeclampsia (n=15), fetal growth restriction (n=3), or other severe pregnancy complications (n=4), including one case of fulminant chorioamnionitis (**Figure 1**). After these exclusions, 46 pregnancies (in 45 women) with sPTB before 34 gw were included. Of the included pregnancies, 23 were complicated with PTL and 23 with PPROM. There were 17 parous women included, seven of those had previously experienced a preterm birth <37 gw, either spontaneous or induced. There were four cases of suspected chorioamnionitis and two other women with positive vaginal cultivation for Group B streptococci.

As controls, non-smoking, healthy women aged 18-42, with a body mass index (BMI) 18-30, with normal pregnancies and no history of obstetric complications, defined as no adverse outcomes in either mother or child in either present or previous pregnancies, were included. An additional inclusion criterion for the control group was that blood samples from both the first and the second trimesters had been collected. *A priori* analysis showed that a sample size of 70 women was sufficient to detect a 40% decrease or increase in an immunological parameter (CXCL10) with a power of 80% and an *alpha* value of 0.05. Hence, 46 women were included as controls. The controls were matched to the cases regarding parity. The selection of controls was based on chronological order regarding the enrollment in the pregnancy biobank, *i.e.*, when a case was identified in the pregnancy biobank, the next woman on the enrollment list subsequent to that case and who fulfilled the inclusion criteria for controls, was included as a control. None of the women in the study received any progesterone treatment.

### Determination of plasma protein biomarkers by using the proximity extension assay

EDTA tubes were used to obtain plasma. After 30-60 minutes the tubes were centrifuged at 2500g for ten minutes and then immediately frozen in aliquots at -70°C until use.

Plasma samples were sent to the SciLifeLab Affinity Proteomics at Uppsala University, Sweden, for analysis of two panels of 92 proteins (the Olink Target 96 Inflammation panel https://olink.com/products-services/target/inflammation/ and the Olink Target 96 Cardiovascular II panel https://olink.com/products-services/target/cardiometabolic-panel/) (Olink Bioscience, Uppsala, Sweden). In brief, one µL of plasma sample was incubated with oligonucleotide-labeled antibodies, which bound pair-wise to two epitopes of each target protein. Next, proximity-dependent DNA polymerization formed a new PCR target sequence if the two antibodies were in close proximity. Real-time PCR (Fluidigm^®^ BioMark™HD System) was then used to detect, amplify, and quantify the PCR target. The data is presented as Normalized Protein eXpression (NPX) values, which are arbitrary units on a Log2 scale, to minimize intra- and inter-assay variation, and used for relative quantification only, inferring that NPX values for two different analytes are not comparable. Due to partial overlaps in the panels, 177 unique proteins were analyzed. Proteins that were measurable above the limit of detection (LOD) in at least 30% of samples (n=158) were included for further statistical analysis. Values below LOD were substituted with LOD-1.

### Statistical analyses

Non-parametric Kruskal-Wallis analysis of variance was used for continuous parameters when comparing more than two groups. For categorical variables, a Chi-2 test or Fisher’s exact test was used when appropriate. P-values <0.05 were considered significant. To evaluate the association of analyzed proteins with sPTB before 34 gw, multivariate logistic regression analysis was performed. Initially, proteins with a p-value <0.1 obtained by a Mann-Whitney *U*-test were accepted for further analysis. Finally, those with p<0.05 in the multivariate logistic regression analysis were included.

The study population was randomly split into two groups of cases and their respective controls. To reduce the risk of chance associations, only proteins that differed significantly (p<0.1) between cases and controls in both random groups in the Mann-Whitney *U*-test were included in the multivariate logistic regression analysis after transformation to quartiles. Receiver operating characteristic curves using the results from the multivariate logistic regression analyses were then defined. The software Statistica v.13.5.0.17 (TIBCO Software Inc., Palo Alto, CA, USA) and MedCalc Statistical Software v. 19.6 (MedCalc Software Ltd, Ostend, Belgium; https://www.medcalc.org/) was used for all statistical analyses.

## Results

### Maternal characteristics

There were no significant differences between women with sPTB <34 gw and women with term birth regarding age, BMI, smoking, parity, or gestational age when the blood samples were taken. There was a significant difference in gestational age at delivery comparing cases (median gw 32 (range 23-34)) and controls (gw 40 (37-42)). The median gestational age at the first trimester sampling was 11 gw (range 7-16) and at the second trimester sampling it was 25 gw (range 23-30) (**Table 1**).

**Table 1:** Characteristics of the study groups of women with spontaneous preterm birth before 34 gestational weeks (cases) and women with normal pregnancy and term birth (controls).

Variable	Cases	Controls	Total	Diff (p)
**N**	46	46	92	
Age (years)				
**Median (range)**	31 (21-40)	30 (24-18)	30 (21-40)	0.319
Body mass index in early pregnancy (kg/m2)				
**Median (range)**	25 (16-40)	24 (18-29)	24 (16-40)	0.077
Smoking (n; %)				
**No**	45 (97.8)	46 (100)	91.0 (98.9)	1.000
**Yes**	1 (2.2)	0		
Gestational week at first trimester sampling				
**Mean (SD)**	10.7 (1.6)	10.8 (1.8)	10.8 (1.7)	
**Median (range)**	11 (7-15)	11 (7-16)	11 (7-16)	0.908
Gestational week at second trimester sampling				
**Mean (SD)**	26.4 (2.1)	26.3 (2.2)	26.4 (2.1)	
**Median (range)**	26 (23-30)	25 (24-30)	25 (23-30)	0.767
Gestational week at delivery				
**Mean (SD)**	30.0 (3.9)	40.2 (1.2)	35.1 (5.9)	
**Median (range)**	32 (23-34)	40 (37-42)	36 (23-42)	<0.001
Mode of delivery (n; %)				
**Vaginal delivery**	34 (73.9)	44 (95.7)	78 (84.8)	
**Cesarean section**	12 (26.1)	2 (4.3)	14.0 (15.2)	0.007

A Kruskal-Wallis analysis of variance was implemented for continuous variables and a Chi-2 test or Fisher’s exact test if appropriate for categorical variables.

### Inflammation-associated proteins in relation to gestational age at delivery

In the first trimester, ten different inflammation-associated proteins were significantly higher (p<0.05) among women with sPTB before 34 weeks than among women with term delivery, whereas two proteins were significantly lower in women with sPTB than in the control group (**Table 2**). In the second trimester, plasma samples were available from 31 of the 46 women with sPTB before 34 gw. The levels of 39 unique proteins were significantly higher (p<0.05) in cases than in controls (**Table 3**). After the random division of the study population, which was done to avoid the problem of mass significance (described in Methods), another analysis by Mann-Whitney *U*-test was performed to reduce chance associations. Only one protein differed (p<0.1) between cases and controls in the first trimester in both random groups (shown by p* in **Table 2**). In the second trimester, the levels of 12 proteins differed (p<0.1) between cases and controls in both random groups (shown by p* in **Table 3**). All protein levels were higher in women with sPTB than in women with term delivery. The levels of all proteins in the first and second trimesters are shown in **Supplementary Table 1**.

**Table 2:** Plasma proteins that significantly (p<0.05) differed in the first trimester between women with preterm birth before 34 gestational weeks (cases) and women with normal pregnancies and term births (controls).

				Trim1			
Assay	Uniprot ID	Panel	<LOD (%)	Cases	Controls	P	p*
N				46	46		
CCL19	Q99731	1	0%	8.38 (8.09-8.69)	8.11 (7.93-8.44)	0.048	
CD40	P25942	1	0%	10.66 (10.48-10.82)	10.48 (10.28-10.63)	0.001	
CDCP1	Q9H5V8	1	0%	2.37 (2.04-2.64)	2.20 (1.96-2.44)	0.039	
HAOX1	Q9UJM8	2	1%	4.77 (4.04-6.28)	4.19 (3.66-5.08)	0.035	
HGF	P14210	1	0%	8.37 (8.10-8.59)	8.20 (8.02-8.36)	0.011	0.065
LOX-1	P78380	2	1%	7.57 (7.18-7.73)	7.24 (6.97-7.53)	0.035	
M-CSF	P09603	1	0%	9.84 (9.70-10.10)	9.74 (9.63-9.91)	0.019	
OSM	P13725	1	0%	4.88 (4.18-5.68)	4.46 (3.88-5.04)	0.037	
RAGE	Q15109	2	1%	13.05 (12.88-13.17)	13.15 (12.99-13.35)	0.016	
REN	P00797	2	1%	7.91 (7.72-8.20)	8.10 (7.92-8.31)	0.015	
TNFSF14	O43557	1	0%	3.46 (3.17-3.80)	3.33 (3.07-3.54)	0.027	
VEGF-A	P15692	1	0%	9.90 (9.79-10.10)	9.81 (9.70-9.90)	0.018	

A Proximity Extension Assay was used and median values and quartiles (Q1-Q3) of NPX values are shown. NPX values are in a log2 scale; therefore, a difference of 1 NPX represents a doubling of the protein concentration. A non-parametric Mann-Whitney U-test was used. p*= maximum p-value in Mann-Whitney U-test after random division of the case-control dyads into two groups.

LOD, Limit of detection. Panel 1=Olink Target 96 Inflammation panel. Panel 2=Olink Target 96 Cardiovascular II panel.

**Table 3:** Plasma proteins that significantly (p<0.05) differed in the second trimester between women with preterm birth before 34 gestational weeks (cases) and women with normal pregnancies and term births (controls).

				Trim2			
Assay	Uniprot ID	Panel	<LOD (%)	Cases	Controls	P	p*
N				31	46		
ACE2	Q9BYF1	2	1%	3.82 (3.64-4.17)	3.59 (3.51-3.83)	0.011	
AGRP	O00253	2	1%	5.49 (5.30-5.84)	5.31 (5.08-5.53)	0.016	
ANG-1	Q15389	2	1%	7.31 (6.99-8.01)	7.05 (6.51-7.54)	0.043	
CA5A	P35218	2	15%	3.39 (2.90-3.91)	2.92 (2.51-3.41)	0.005	
CASP-8	Q14790	1	53%	1.08 (-0.01-1.21)	-0.01 (-0.01-1.07)	0.036	
CCL2	P13500	1	0%	10.39 (10.19-10.84)	10.26 (10.03-10.52)	0.050	
CCL20	P78556	1	0%	5.44 (4.97-5.94)	5.07 (4.66-5.60)	0.036	
CCL3	P10147	1	0%	4.12 (3.85-4.53)	3.82 (3.64-4.13)	0.003	0.064
CCL3	P10147	2	1%	6.05 (5.72-6.54)	5.73 (5.57-6.08)	0.014	
CCL4	P13236	1	0%	5.42 (4.98-5.72)	5.12 (4.89-5.32)	0.015	
CD40	P25942	1	0%	10.58 (10.42-10.84)	10.28 (10.14-10.56)	0.001	0.036
CXCL10	P02778	1	0%	8.61 (8.12-9.05)	8.18 (7.83-8.49)	0.002	0.076
CXCL11	O14625	1	0%	7.81 (7.28-8.46)	7.26 (6.83-7.88)	0.008	
CXCL8	P10145	1	0%	3.67 (3.32-3.97)	3.23 (3.12-3.64)	0.014	
CXCL9	Q07325	1	0%	6.04 (5.74-6.47)	5.74 (5.43-6.23)	0.013	
DCN	P07585	2	1%	3.90 (3.61-4.03)	3.71 (3.56-3.93)	0.036	
FGF-21	Q9NSA1	1	23%	3.21 (2.12-4.30)	2.60 (0.84-3.27)	0.027	
FGF-21	Q9NSA1	2	1%	4.97 (3.75-5.86)	4.27 (2.93-4.82)	0.030	
Flt3L	P49771	1	0%	8.98 (8.58-9.14)	8.65 (8.42-8.87)	0.005	
Gal-9	O00182	2	1%	7.56 (7.38-7.86)	7.43 (7.32-7.57)	0.017	
HAOX1	Q9UJM8	2	1%	6.23 (4.53-6.94)	5.13 (4.58-5.96)	0.041	
HGF	P14210	1	0%	8.63 (8.38-8.84)	8.42 (8.15-8.63)	0.023	
IL-10	P22301	1	0%	3.59 (3.45-3.93)	3.42 (3.17-3.57)	0.001	0.096
IL-10RB	Q08334	1	0%	5.85 (5.75-6.00)	5.75 (5.58-5.95)	0.035	
IL-18	Q14116	1	0%	9.06 (8.53-9.44)	8.66 (8.48-8.92)	0.009	
IL-18	Q14116	2	1%	9.06 (8.53-9.44)	8.66 (8.48-8.92)	0.019	
IL-18R1	Q13478	1	0%	8.25 (7.63-8.47)	7.89 (7.69-8.16)	0.047	
IL-1ra	P18510	2	1%	5.18 (4.85-6.22)	4.79 (4.63-5.00)	<0.001	0.015
IL-6	P05231	1	2%	2.80 (2.47-3.32)	2.37 (2.16-2.66)	<0.001	0.012
IL-6	P05231	2	1%	3.14 (2.82-3.64)	2.71 (2.48-2.90)	<0.001	0.007
LAP TGF-beta-1	P01137	1	0%	7.76 (7.41-7.96)	7.50 (7.24-7.76)	0.013	
LOX-1	P78380	2	1%	8.69 (8.18-8.95)	8.27 (7.91-8.51)	0.003	0.073
M-CSF	P09603	1	0%	10.14 (10.01-10.26)	9.95 (9.86-10.07)	<0.001	0.013
MMP-1	P03956	1	0%	9.24 (8.23-9.98)	8.43 (7.69-9.06)	0.009	
OPG	O00300	1	0%	10.79 (10.40-11.18)	10.38 (10.13-10.88)	0.016	
PARP-1	P09874	2	1%	3.45 (3.24-3.67)	3.17 (2.91-3.42)	0.002	0.041
RANK	Q9Y6Q6	2	1%	5.87 (5.60-6.14)	5.52 (5.43-5.70)	0.001	0.053
S100A12	P80511	1	0%	1.61 (1.24-2.02)	1.29 (1.05-1.56)	0.010	
SPON2	Q9BUD6	2	1%	8.36 (8.28-8.40)	8.29 (8.21-8.36)	0.041	
TRAIL	P50591	1	0%	7.44 (7.21-7.62)	7.26 (7.07-7.46)	0.025	
TRAIL-R1	O00220	2	1%	3.11 (2.84-3.31)	2.94 (2.83-3.11)	0.033	
TRAIL-R2	O14763	2	1%	5.97 (5.75-6.18)	5.84 (5.67-5.98)	0.042	
VEGF-A	P15692	1	0%	10.20 (10.01-10.31)	10.05 (9.92-10.14)	0.002	0.047

Among the cases, second trimester samples were available for 31 of the 46 women. A Proximity Extension Assay was used and median values and quartiles (Q1-Q3) of NPX values are shown. NPX values are in a log2 scale; therefore, a difference of 1 NPX represents a doubling of the protein concentration. A non-parametric Mann-Whitney U-test was used. p*= maximum p-value in Mann-Whitney U-test after random division of the case-control dyads into two groups.

LOD, Limit of Detection; Percentages indicate <LOD for sampling occasions in both the first and the second trimesters. Panel 1=Olink Target 96 Inflammation panel. Panel 2=Olink Target 96 Cardiovascular II panel.

After transformation of the protein data into quartiles, a multivariate logistic regression analysis was performed including all proteins that differed between cases and controls at a level of p<0.1. In the first trimester, higher levels of hepatocyte growth factor (HGF) were associated with sPTB<34 gw (odds ratio (OR) 1.49 (1.03-2.15). In the second trimester, higher levels of interleukin (IL)-10 (OR 2.15 (1.18-3.92), IL-6 (OR 2.59 (1.34-4.99), and receptor activator of nuclear factor κB (RANK) (OR 2.18 (1.26-3.77) were associated with sPTB <34 gw (**Table 4**).

**Table 4:** Plasma proteins associated with spontaneous preterm birth before 34 gestational weeks measured by a Proximity Extension Assay in the first and second trimesters, respectively.

First trimester	All	Preterm birth			
Variable	N	n	%	OR (95% CI)	p
HGF (P14210)					
≤8.02	20	8	40.0	1.00	
8.03-8.19	16	6	37.5	1.49 (1.03-2.15)	
8.20-8.36	19	7	36.8	2.22 (1.07-4.62)	
>8.36	37	25	67.6	3.31 (1.10-9.92)	0.033

Second trimester	All	Preterm birth			
Variable	N	n	%	OR (95% CI)	p
IL-10 (P22301)					
≤3.17	13	2	15.4	1.00	
3.18-3.44	17	5	29.4	2.15 (1.18-3.92)	
3.45-3.57	16	6	37.5	4.64 (1.40-15.4)	
>3.57	31	18	58.1	10.0 (1.66-60.3)	0.013
IL-6 (P05231)					
≤2.16	13	2	15.4	1.00	
2.17-2.37	14	2	14.3	2.59 (1.34-4.99)	
2.38-2.67	20	8	40.0	6.71 (1.81-24.9)	
>2.67	30	19	63.3	17.4 (2.43-124)	0.005
RANK (TNFRSF11A) (Q9Y6Q6)					
≤5.43	14	4	28.6	1.00	
5.44-5.52	13	2	15.4	2.18 (1.26-3.77)	
5.53-5.69	17	5	29.4	4.76 (1.59-14.2)	
>5.69	31	20	64.5	10.4 (2.01-53.6)	0.006

The protein data was transformed into quartiles and a multivariate logistic regression analysis was performed.

### Prediction accuracy of PTB before 34 gw based on protein combinations

**Figure 2:**
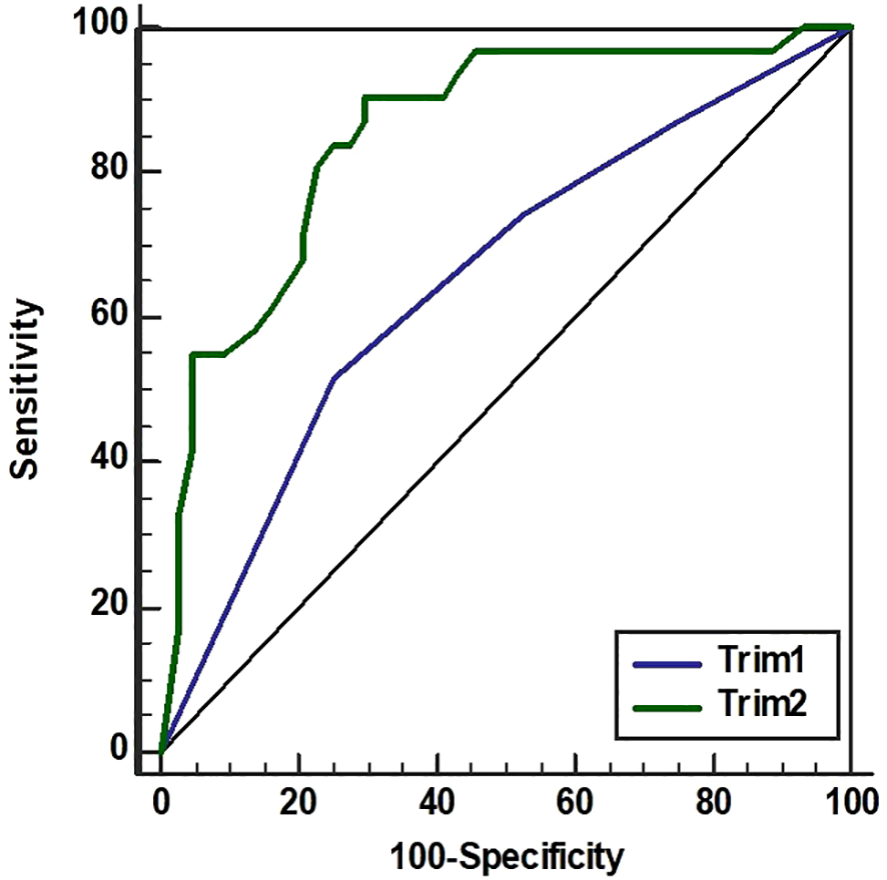
Index for spontaneous preterm birth before 34 gestational weeks. Receiver operating characteristic curves resulting from the multivariate logistic regression analyses and their respective areas under the curve for the first trimester (Trim1, blue line) and the second trimester (Trim 2, green line). Proteins were measured by Proximity Extension Assay in plasma longitudinally sampled from women with sPTB before 34 gestational weeks (n=46 in Trim1, n=31 in Trim2) compared with women with delivery at term (n=46 in both trimesters). In Trim1 the protein HGF was included. In Trim2, IL-6, IL-10, and RANK were included.

Receiver operating characteristic curves using the results from the multivariate logistic regression analyses were defined creating prediction models for sPTB before 34 gw. The area under the curve (AUC) for the prediction models of the respective blood sampling time points was 0.653 (95% CI 0.534-0.759) in the first trimester and 0.854 (95% CI 0.754-0.925) in the second trimester (**Figure 2**). The proteins in the prediction models were HGF in the first trimester and IL-10, IL-6, and RANK in the second trimester.

## Discussion

In this case-control study including 45 women with 46 spontaneous preterm deliveries <34 gw and 46 control women with normal pregnancies, the plasma HGF levels in the first trimester were able to predict sPTB <34 gw with an AUC of 0.653. However, a combination of the plasma proteins IL-6, IL-10 and RANK sampled in the second trimester was substantially better at predicting sPTB <34 gw with an AUC of 0.854. It is likely that evolving pathology during the course of pregnancy results in higher accuracy of prediction at a later gestational age.

In relation to previous work regarding the prediction of sPTB, our model based on plasma proteins in the second trimester is reasonably good. Ngo et al. (26) presented an AUC of 0.81 based on transcripts in maternal blood in a rather small (n=23) validation cohort of women with a high risk of PTB. In a study by Saade et al. (23), based on combinations of circulating proteins in serum samples collected at 17-28 gw, the AUC was 0.75. Moreover, Mavreli et al. (22) showed good predictive ability with AUC ranging from 0.82-0.96 when investigating first trimester proteomics using liquid chromatography tandem mass spectrometry and subsequent enzyme-linked immunosorbent assay. However, the focus in this study was sPTB at 32-36 gw, not sPTB before 34 gw. In models built from plasma proteomic data from samples collected at 27-33 weeks of gestation in Tarca et al. (20) the presented AUC was 0.76, whereas, in the same study the AUC based on a predictive model of transcriptomic data was 0.6. Altogether, this might imply that proteomics may be a more sensitive method for the prediction of sPTB than transcriptomics. Yet, the area of transcriptomic discovery and validation of possible predictive markers and subsequent pathway analysis is a promising field, as shown by Weiner et al. (21). Also, the metabolomic approach to the prediction of PTB is interesting with several studies presenting prediction models with AUC 0.82-0.92 (15, 24, 25).

Another predictive model with high accuracy was presented by Cantonwine et al. (27) using different combinations of circulating microparticles where the top 20 combinations predicted sPTB before 34 gw, with an AUC ranging from 0.861 to 0.892. The method used by us offers a fast and more feasible way of measuring circulating biomarkers without the need for extraction of RNA or microparticles, or the need of preparation of samples for mass spectrometry, with potential for pre-analytical errors. Furthermore, the current surge in PEA-based biomarker discovery in different fields could accelerate the development of bedside tests (30). However, according to our results, it seems difficult to reliably predict PTB <34 gw in the first trimester based on inflammation-associated plasma proteins. We present an AUC of a mere 0.653 although as many as 177 unique proteins were analyzed.

The proteins that were included in our prediction models have previously been associated either with PTB or various other pregnancy complications, strengthening the findings of our study. HGF is a growth factor that plays an important role in tissue regeneration and repair in various organ systems (31). Placental HGF has been reported to be strongly expressed in the villous syncytium, extravillous trophoblast, and amnionic epithelium (32). In umbilical vein blood, an increase of HGF levels throughout normal pregnancies has been found (33), although there might instead be an inverse correlation between activated HGF and gestational age (34). Furthermore, in healthy women with normal pregnancies HGF in maternal plasma has been shown to be upregulated in the third trimester and then downregulated postpartum (35). Altogether, these findings suggest that HGF plays a significant role in pregnancy. It is plausible that HGF is also important in pregnancy complications, even if results are somewhat contradictory. Significantly elevated levels of HGF maternal serum in pregnancies complicated by preeclampsia have been found (36), whereas in umbilical cord blood no association was found between HGF levels and pregnancies complicated by pregnancy-induced hypertension (33). Regarding small-for-gestational age fetuses, HGF has been found to be increased in maternal plasma (37); however, no such difference was found in cord blood (33). To the best of our knowledge, no associations have previously been found regarding maternal plasma levels of HGF and PTB. However, higher levels of activated HGF have been found in the cord blood of preterm infants compared to term neonates, although total HGF levels did not differ (34).

IL-6 and IL-10 are both well-known cytokines and crucial for inflammatory and anti-inflammatory responses, respectively (38, 39). These interleukins have previously been associated with PTB after PTL in samples obtained from blood (40), cervicovaginal fluid (41), and amniotic fluid (42). Regarding the prediction of PTB in early pregnancy, IL-6 is one of the most frequently studied biomarkers (43, 44), but has thus far not been shown to be of clinical use on its own. The association between IL-10 and PTB using multiplex analysis has also been studied repeatedly, but has not yet been convincingly shown to have a real value as a predictor for PTB (45).

The receptor RANK and its ligand RANKL are necessary for osteoclastogenesis and bone homeostasis but are also essential for several features of the immune system, including the development of the thymic medulla, lymph nodes and microfold cells in the intestine. Hence, RANKL/RANK are also important for T cell function and control (46, 47). RANKL also might have a role in maternal-fetal tolerance, since it has been shown that polarization of decidual macrophages toward an M2 phenotype is induced by RANKL secreted from human embryonic trophoblasts and maternal decidual stromal cells (48). Furthermore, the possible importance of the RANKL-RANK system in pregnancy immunology and pregnancy complications is illustrated by findings of lower levels of RANKL on decidual stromal cells and of RANK on decidual γδ T cells in decidua from patients with recurrent spontaneous miscarriage compared to normal pregnancy (49). RANKL is also potentially involved in other pregnancy complications; infants born preterm after pregnancies complicated by early-onset preeclampsia have lower RANKL serum concentrations at birth than full-term and preterm-born babies after normotensive pregnancies (50). Finally, RANKL might be associated with gestational diabetes mellitus, as lower levels of RANKL have been found in women with gestational diabetes mellitus compared to women with normal pregnancies. HGF was also investigated in the same study, although no association was found with gestational diabetes mellitus (51). Regarding RANKL-RANK, we have not found any previous reports on the association with PTB, so this also is a new finding.

In our study, preeclampsia and fetal growth restriction were exclusion criteria. Thus, the demonstrated association between HGF and PTB, as well as between RANK and PTB, can be regarded as a true association with PTB and is not affected by placental pathology. In **Table 5** a brief summary of previous findings related to pregnancy and pregnancy complications regarding HGF, IL-6, IL-10, RANK/RANKL is presented.

**Table 5:** Brief overview of previous findings related to pregnancy and pregnancy complications regarding HGF, IL-6, IL-10, RANK/RANKL.

Protein	*Biological function*	Findings related to pregnancy or pregnancy complications	Sample type	Ref.
**HGF**	*Growth factor, important role in tissue regeneration and repair in various organ systems.*			(31)
		Strongly expressed in the villous syncytium, extravillous trophoblast, and amnionic epithelium.	Placenta	(32)
		Upregulated in the third trimester, downregulated postpartum in healthy women with normal pregnancies.	Maternal plasma	(35)
		Increase of levels throughout normal pregnancies, no association with pregnancy-induced hypertension, no association with pregnancies with small-for-gestational age fetuses.	Umbilical vein serum	(33)
		Elevated levels in preeclamptic pregnancies.	Maternal serum	(36)
		Increased in pregnancies with small-for-gestational age fetuses.	Maternal plasma	(37)
		Activated HGF higher in preterm than in term neonates, no difference in total levels.	Cord blood	(34)
**IL-6**	*Crucial for inflammatory responses.*			(38)
		Higher levels in PTL with PTB <37 gw compared to PTL with term birth.	Maternal serum	(40)
		High levels associated with PTB within 7 days after PTL.	Cervicovaginal fluid	(41)
		Elevated in PTL with PTB and intraamniotic infection.	Amniotic fluid	(42)
		Repeatedly shown associations with PTB.	Miscellaneous (systematic reviews)	(43, 44)
**IL-10**	*Crucial for anti-inflammatory responses and immune tolerance.*			(39)
		No significant difference in PTL with PTB <37 gw compared to PTL with term birth.	Maternal serum	(40)
		Repeatedly shown associations with PTB.	Miscellaneous (systematic review)	(45)
**RANK/RANKL**	*Necessary for osteoclastogenesis and bone homeostasis, essential for the immune system (development of the thymic medulla, lymph nodes and microfold cells in the intestine, important for T cell function and control).*			(46)
		Induction of polarization of decidual macrophages toward an M2 phenotype; role in maternal-fetal tolerance.	Human embryonic trophoblasts, maternal decidual stromal cells	(48)
		Lower levels in recurrent spontaneous miscarriages compared to normal pregnancies.	Decidua	(49)
		Lower in preterm births after pregnancies with early-onset preeclampsia than in full-term births in normotensive pregnancies.	Neonatal serum	(50)

PTL, preterm labor; PTB, preterm birth; gw, gestational week.

Various pathogens have been linked to PTB (52). It is reasonable to assume that several cases of sPTB in our study was caused by a subclinical infection. Information from the medical files presents four cases of suspected, but not verified, chorioamnionitis. The clinical guidelines during the study period did not include amniocentesis for microbiological PCR-testing or cultivation. Furthermore, two women were cultivation positive for Group B streptococci in the vagina. There is no further information available whether these findings were assessed as clinically relevant or not. It can be speculated that the inflammatory biomarkers might hold greater discriminatory power for a subgroup of PTBs caused by infectious agents. However, this theory is not possible to test, as the proportion of women with clinically suspected infections in our case-control cohort was small, conceivably six women of in total 46 with sPTB.

The strengths of this study are several. We conducted a population-based study, in which 95% of the pregnant women in this part of Sweden were offered participation in the study. Furthermore, the study population overall displayed a low risk of PTB, which provides better conditions to create a prediction model for sPTB that is applicable to pregnant women in general, instead of being aimed at a group of pregnant women with a previously known high risk of PTB. Also, the participating individual women underwent repeated blood samplings, providing a comprehensive description of the analyzed plasma proteins. Additionally, we studied a large number of plasma proteins (n=177) using a Proximity Extension Assay, which is a high-sensitivity and high-specificity method, in total offering top conditions for the reliable detection of proteins and the creation of a prediction model. At present this technology is not available in standard hospital laboratory settings. However, since the technique is PCR-based it is possible that, in the near future, it might be used in standard laboratory practice.

This study also has some potential limitations. Although the total incidence of PTB before 34 gw in our pregnancy biobank (107/7,095 = 1.5%) is comparable to the incidence in the Swedish population (53), the cohort is relatively small regarding the cases with sPTB (n=46). Seven women who had experienced PTB previously were included in the case group. It cannot be ruled out that the result could have been affected if these women were excluded. We have regarded PTL and PPROM as one clinical entity of sPTB, even though the two conditions of PTL and PPROM are different phenotypically and may well have different underlying mechanisms. However, the aim in this work was to predict sPTB <34 gw regardless of the underlying mechanism, which is an argument in favor of investigating PTL and PPROM as one entity. As controls, we included healthy non-smoking women with normal BMI and previous, and present, normal pregnancies. The controls were matched to the cases only regarding parity. Thus, the control group may not completely represent the population in general, but rather a group with fewer health problems and potentially a lower background level in inflammatory markers, possibly exaggerating the difference between cases and controls. However, there were no major differences in smoking and BMI across groups. Another potential limitation is that information on the ethnicity of the women included in the study was not available since this was not registered in the medical files of the women. However, the vast majority of the patients at the two antenatal clinics were Caucasian.

In conclusion, in this case-control study including 45 women with 46 spontaneous preterm deliveries <34 gw and 46 women with normal pregnancies and term births, a combination of inflammation-associated plasma proteins (IL-6, IL-10, and RANK) from the second trimester of pregnancy showed a good predictive ability (AUC 0.854) regarding sPTB before 34 gw, suggesting it could be a valuable supplement for assessment of clinical risk of sPTB. Future studies are needed to validate these findings. Although our method is consistent with a low-risk population, well-defined cases of PTB, and a high number (n=177) of plasma proteins analyzed with a high-sensitivity and high-specificity method, the predictive ability in the first trimester regarding sPTB was unsatisfactory (AUC 0.653) from a clinical point of view. Future studies should thus involve larger cohorts with analyses of more proteins, and might possibly also combine different types of biomarkers, such as proteomics with transcriptomics.

## Data availability statement

The raw data supporting the conclusions of this article will be made available by the authors, without undue reservation.

## Ethics statement

The studies involving humans were approved by the Regional Ethical Review Board in Linköping, Sweden (2010/296-31 and 2019-00424). The studies were conducted in accordance with the local legislation and institutional requirements. The participants provided their written informed consent to participate in this study.

## Author contributions

MS: Conceptualization, Data curation, Formal analysis, Funding acquisition, Investigation, Methodology, Writing – original draft, Writing – review & editing. JR: Conceptualization, Formal analysis, Investigation, Methodology, Project administration, Resources, Writing – review & editing. LB: Data curation, Formal analysis, Investigation, Methodology, Resources, Software, Supervision, Validation, Writing – review & editing. GB: Conceptualization, Methodology, Supervision, Validation, Visualization, Writing – review & editing. SH: Formal analysis, Investigation, Methodology, Resources, Writing – review & editing. MB: Conceptualization, Investigation, Methodology, Project administration, Supervision, Validation, Visualization, Writing – review & editing. MJ: Conceptualization, Methodology, Resources, Supervision, Validation, Visualization, Writing – review & editing. JE: Conceptualization, Formal analysis, Funding acquisition, Investigation, Methodology, Resources, Supervision, Validation, Visualization, Writing – original draft, Writing – review & editing.
