# Supplementary material for: Early prediction of spontaneous preterm birth before 34 gestational weeks based on a combination of inflammation-associated plasma proteins
Source: Front Immunol. 2024 Jul 15;15:1415016. doi: 10.3389/fimmu.2024.1415016 (PMC11284114; doi:10.3389/fimmu.2024.1415016)
Supplement: Supplementary file 1 [file Table_1.docx]

**Supplementary table 1.** Proteins and their Uniprot IDs analyzed by a Proximity Extension Assay (alternative protein names in brackets). Median values, quartiles (Q1-Q3), and P-values below 0.1 are shown. NPX values are in a log2 scale; therefore, a difference of 1 NPX represents a doubling of protein concentration. The study population consisted of women with preterm birth before 34 gestational weeks (cases) and women with normal pregnancies and term birth (controls). A non-parametric Mann-Whitney *U*-test was used.

|  |  |  |  |  | **Trim1** | | |  | **Trim2** | | |
| --- | --- | --- | --- | --- | --- | --- | --- | --- | --- | --- | --- |
| **Assay** | **Uniprot ID** | **Panel*** | **LOD** | **<LOD (%)** | **Cases** | **Controls** | **P** |  | **Cases** | **Controls** | **P** |
| N |  |  |  |  | 46 | 46 |  |  | 31 | 46 |  |
| 4E-BP1 | Q13541 | 1 | 1.28 | 0% | 7.43 (6.84-7.96) | 7.19 (6.77-7.61) |  |  | 7.46 (6.62-8.09) | 7.05 (6.63-7.75) |  |
| ACE2 | Q9BYF1 | 2 | 1.44 | 1% | 3.45 (3.15-3.87) | 3.41 (3.21-3.55) |  |  | 3.82 (3.64-4.17) | 3.59 (3.51-3.83) | 0.011 |
| ADA | P00813 | 1 | 0.96 | 0% | 4.87 (4.72-5.20) | 4.82 (4.63-5.01) |  |  | 4.81 (4.59-5.24) | 4.71 (4.51-4.93) |  |
| ADAM-TS13 | Q76LX8 | 2 | 1.28 | 1% | 5.38 (5.28-5.43) | 5.34 (5.28-5.42) |  |  | 5.28 (5.19-5.39) | 5.29 (5.21-5.40) |  |
| ADM | P35318 | 2 | 2.12 | 1% | 8.42 (8.24-8.64) | 8.33 (8.22-8.50) |  |  | 9.58 (9.29-9.79) | 9.50 (9.27-9.65) |  |
| AGRP | O00253 | 2 | 1.30 | 1% | 5.27 (5.08-5.49) | 5.21 (4.96-5.47) |  |  | 5.49 (5.30-5.84) | 5.31 (5.08-5.53) | 0.016 |
| AMBP | P02760 | 2 | 1.32 | 1% | 7.80 (7.70-7.91) | 7.81 (7.74-7.89) |  |  | 7.83 (7.74-7.97) | 7.81 (7.73-7.89) |  |
| ANG-1 | Q15389 | 2 | 0.18 | 1% | 7.38 (6.81-7.94) | 7.21 (6.71-7.65) |  |  | 7.31 (6.99-8.01) | 7.05 (6.51-7.54) | 0.043 |
| ARTN | Q5T4W7 | 1 | 1.06 | 61% | 0.06 (0.06-0.06) | 0.06 (0.06-0.06) |  |  | 1.43 (1.14-1.73) | 1.28 (0.06-1.63) |  |
| AXIN-1 | O15169 | 1 | 1.07 | 1% | 2.76 (2.11-3.75) | 2.65 (2.13-3.02) |  |  | 2.78 (2.36-3.18) | 2.62 (1.98-3.15) |  |
| Beta-NGF | P01138 | 1 | 1.02 | 87% |  |  |  |  |  |  |  |
| BMP-6 | P22004 | 2 | 1.36 | 1% | 4.47 (4.26-4.80) | 4.55 (4.42-4.66) |  |  | 4.29 (3.43-4.58) | 4.09 (3.75-4.37) |  |
| BNP | P16860 | 2 | 1.38 | 27% | 2.15 (1.79-2.46) | 2.09 (1.73-2.66) |  |  | 1.58 (0.27-1.78) | 1.51 (0.19-1.85) |  |
| BOC | Q9BWV1 | 2 | 1.14 | 1% | 3.65 (3.45-3.81) | 3.67 (3.57-3.86) |  |  | 3.66 (3.45-3.87) | 3.61 (3.53-3.78) |  |
| CA5A | P35218 | 2 | 2.07 | 15% | 2.62 (2.23-2.95) | 2.31 (0.99-2.71) |  |  | 3.39 (2.90-3.91) | 2.92 (2.51-3.41) | 0.005 |
| CASP-8 | Q14790 | 1 | 0.99 | 53% | -0.01 (-0.01-1.11) | -0.01 (-0.01-1.10) |  |  | 1.08 (-0.01-1.21) | -0.01 (-0.01-1.07) | 0.036 |
| CCL11 (Eotaxin-1) | P51671 | 1 | 1.45 | 0% | 5.47 (5.18-5.69) | 5.52 (5.27-5.79) |  |  | 5.13 (4.90-5.34) | 5.16 (4.93-5.38) |  |
| CCL13 (MCP-4) | Q99616 | 1 | 2.13 | 0% | 11.92 (11.54-12.34) | 11.84 (11.60-12.41) |  |  | 11.57 (11.18-12.04) | 11.48 (11.03-11.70) |  |
| CCL17 (TARC) | Q92583 | 2 | 1.85 | 1% | 7.57 (7.30-7.95) | 7.63 (7.22-8.13) |  |  | 6.95 (6.60-7.55) | 6.95 (6.66-7.82) |  |
| CCL19 (MIP-3beta) | Q99731 | 1 | 1.10 | 0% | 8.38 (8.09-8.69) | 8.11 (7.93-8.44) | 0.048 |  | 8.17 (7.65-8.47) | 7.84 (7.49-8.17) |  |
| CCL2 (MCP-1) | P13500 | 1 | 1.05 | 0% | 10.45 (10.25-10.59) | 10.46 (10.29-10.66) |  |  | 10.39 (10.19-10.84) | 10.26 (10.03-10.52) | 0.050 |
| CCL20 (MIP-3alpha) | P78556 | 1 | 2.06 | 0% | 5.41 (5.17-5.82) | 5.49 (5.21-5.85) |  |  | 5.44 (4.97-5.94) | 5.07 (4.66-5.60) | 0.036 |
| CCL23 (MIP-3) | P55773 | 1 | 0.98 | 0% | 9.52 (9.28-9.93) | 9.45 (9.21-9.69) |  |  | 9.19 (8.91-9.43) | 9.07 (8.79-9.38) |  |
| CCL25 | O15444 | 1 | 1.44 | 0% | 5.01 (4.73-5.43) | 4.95 (4.70-5.15) |  |  | 5.13 (4.73-5.47) | 4.97 (4.69-5.24) |  |
| CCL28 (MEC) | Q9NRJ3 | 1 | 1.04 | 0% | 2.40 (2.01-2.66) | 2.48 (2.14-2.74) |  |  | 3.87 (3.52-4.46) | 3.83 (3.44-4.22) |  |
| CCL3 (MIP-1alpha) | P10147 | 1 | 1.00 | 0% | 3.78 (3.53-4.06) | 3.66 (3.51-3.79) |  |  | 4.12 (3.85-4.53) | 3.82 (3.64-4.13) | 0.003 |
| CCL3 (MIP-1alpha) | P10147 | 2 | 2.06 | 1% | 5.70 (5.41-5.93) | 5.65 (5.42-5.84) |  |  | 6.05 (5.72-6.54) | 5.73 (5.57-6.08) | 0.014 |
| CCL4 (MIP-1beta) | P13236 | 1 | 1.18 | 0% | 5.16 (4.78-5.35) | 5.00 (4.76-5.23) |  |  | 5.42 (4.98-5.72) | 5.12 (4.89-5.32) | 0.015 |
| CCL7 (MCP-3) | P80098 | 1 | 1.58 | 91% |  |  |  |  |  |  |  |
| CCL8 (MCP-2) | P80075 | 1 | 1.74 | 0% | 7.68 (7.33-8.02) | 7.73 (7.37-8.02) |  |  | 7.49 (7.22-7.84) | 7.39 (7.05-7.66) |  |
| CD244 | Q9BZW8 | 1 | 1.69 | 0% | 6.36 (6.25-6.57) | 6.32 (6.18-6.52) |  |  | 6.30 (6.15-6.46) | 6.29 (6.10-6.42) |  |
| CD4 | P01730 | 2 | 1.28 | 1% | 4.38 (4.28-4.55) | 4.39 (4.25-4.55) |  |  | 4.23 (4.00-4.32) | 4.12 (3.97-4.30) |  |
| CD40 | P25942 | 1 | 1.76 | 0% | 10.66 (10.48-10.82) | 10.48 (10.28-10.63) | 0.001 |  | 10.58 (10.42-10.84) | 10.28 (10.14-10.56) | 0.001 |
| CD40-L | P29965 | 2 | 0.69 | 1% | 3.31 (2.87-4.10) | 3.14 (2.89-3.80) |  |  | 3.20 (2.81-3.86) | 3.23 (2.84-3.52) |  |
| CD5 | P06127 | 1 | 0.28 | 0% | 4.48 (4.24-4.76) | 4.50 (4.33-4.65) |  |  | 4.27 (4.06-4.38) | 4.19 (4.08-4.39) |  |
| CD6 | P30203 | 1 | 0.79 | 0% | 4.64 (4.29-4.95) | 4.64 (4.44-4.75) |  |  | 4.42 (4.21-4.69) | 4.34 (4.12-4.63) |  |
| CD84 | Q9UIB8 | 2 | 2.07 | 1% | 4.39 (4.30-4.64) | 4.38 (4.28-4.55) |  |  | 4.36 (4.23-4.51) | 4.33 (4.19-4.46) |  |
| CD8A | P01732 | 1 | 1.40 | 0% | 10.30 (9.97-10.65) | 10.29 (9.94-10.62) |  |  | 9.71 (9.25-10.10) | 9.83 (9.50-10.13) |  |
| CDCP1 | Q9H5V8 | 1 | -0.37 | 0% | 2.37 (2.04-2.64) | 2.20 (1.96-2.44) | 0.039 |  | 2.87 (2.63-3.09) | 2.70 (2.22-2.95) |  |
| CEACAM8 | P31997 | 2 | 2.02 | 1% | 4.76 (4.40-5.15) | 4.66 (4.28-4.75) |  |  | 4.92 (4.56-5.22) | 4.78 (4.46-5.13) |  |
| CST5 | P28325 | 1 | 0.98 | 0% | 4.41 (4.20-4.61) | 4.33 (4.13-4.57) |  |  | 4.35 (4.10-4.57) | 4.21 (4.09-4.55) |  |
| CTRC | Q99895 | 2 | 2.52 | 1% | 11.02 (10.66-11.48) | 11.01 (10.51-11.23) |  |  | 10.95 (10.67-11.41) | 11.05 (10.51-11.34) |  |
| CTSL1 | P07711 | 2 | 1.21 | 1% | 6.98 (6.85-7.12) | 6.93 (6.82-7.12) |  |  | 6.90 (6.64-7.14) | 6.82 (6.68-6.98) |  |
| CX3CL1 (Fractalkine) | P78423 | 1 | 1.36 | 0% | 5.15 (4.92-5.46) | 5.22 (5.07-5.39) |  |  | 5.01 (4.77-5.24) | 5.03 (4.84-5.29) |  |
| CXCL1 (GRO1) | P09341 | 1 | 1.89 | 0% | 7.77 (7.01-8.47) | 7.95 (7.39-8.47) |  |  | 7.65 (7.29-8.46) | 7.92 (7.55-8.15) |  |
| CXCL1 (GRO1) | P09341 | 2 | 3.46 | 1% | 9.23 (8.57-9.99) | 9.31 (8.79-10.05) |  |  | 9.24 (8.83-9.79) | 9.37 (8.93-9.62) |  |
| CXCL10 (IP-10) | P02778 | 1 | 2.11 | 0% | 8.36 (8.04-8.73) | 8.36 (8.10-8.66) |  |  | 8.61 (8.12-9.05) | 8.18 (7.83-8.49) | 0.002 |
| CXCL11 (I-TAC) | O14625 | 1 | 1.22 | 0% | 7.46 (7.09-8.09) | 7.40 (6.99-7.78) |  |  | 7.81 (7.28-8.46) | 7.26 (6.83-7.88) | 0.008 |
| CXCL5 (CXCL5) | P42830 | 1 | 1.89 | 0% | 9.43 (8.59-10.20) | 9.53 (8.93-10.10) |  |  | 8.90 (8.08-9.71) | 9.17 (8.40-9.79) |  |
| CXCL6 | P80162 | 1 | 0.71 | 0% | 7.46 (7.11-7.95) | 7.62 (7.13-8.03) |  |  | 7.31 (6.97-7.86) | 7.33 (7.11-7.72) |  |
| CXCL8 (IL-8) | P10145 | 1 | 0.71 | 0% | 3.54 (3.33-3.87) | 3.56 (3.27-3.78) |  |  | 3.67 (3.32-3.97) | 3.23 (3.12-3.64) | 0.014 |
| CXCL9 (MIG) | Q07325 | 1 | 1.41 | 0% | 6.13 (5.72-6.54) | 6.02 (5.70-6.31) |  |  | 6.04 (5.74-6.47) | 5.74 (5.43-6.23) | 0.013 |
| DCN | P07585 | 2 | 1.46 | 1% | 4.03 (3.81-4.27) | 4.01 (3.84-4.17) |  |  | 3.90 (3.61-4.03) | 3.71 (3.56-3.93) | 0.036 |
| DECR1 | Q16698 | 2 | 1.99 | 1% | 4.11 (3.48-4.97) | 4.00 (3.63-4.45) |  |  | 4.02 (3.53-4.66) | 3.81 (3.42-4.26) |  |
| Dkk-1 | O94907 | 2 | 0.30 | 1% | 8.71 (8.46-8.96) | 8.64 (8.41-8.75) |  |  | 8.97 (8.76-9.31) | 8.89 (8.70-9.14) |  |
| DNER | Q8NFT8 | 1 | 0.71 | 0% | 8.51 (8.26-8.67) | 8.51 (8.39-8.59) |  |  | 8.33 (8.11-8.53) | 8.33 (8.17-8.44) |  |
| FABP2 | P12104 | 2 | 2.38 | 1% | 7.34 (6.65-7.58) | 7.37 (7.14-7.82) |  |  | 7.61 (6.82-8.26) | 7.54 (7.14-7.97) |  |
| FGF-19 | O95750 | 1 | 1.50 | 0% | 7.90 (7.29-8.47) | 7.77 (7.02-8.20) |  |  | 8.26 (7.53-8.67) | 7.96 (7.52-8.75) |  |
| FGF-21 | Q9NSA1 | 1 | 1.84 | 23% | 4.20 (3.36-5.20) | 4.14 (3.69-4.77) |  |  | 3.21 (2.12-4.30) | 2.60 (0.84-3.27) | 0.027 |
| FGF-21 | Q9NSA1 | 2 | 1.83 | 1% | 4.20 (3.36-5.20) | 4.14 (3.69-4.77) |  |  | 4.97 (3.75-5.86) | 4.27 (2.93-4.82) | 0.030 |
| FGF-23 | Q9GZV9 | 1 | 2.40 | 67% | 1.40 (1.40-1.40) | 1.40 (1.40-1.40) |  |  | 2.67 (2.54-3.07) | 2.65 (2.32-3.15) |  |
| FGF-23 | Q9GZV9 | 2 | 1.76 | 2% | 2.37 (2.24-2.57) | 2.27 (2.12-2.46) |  |  | 2.67 (2.54-3.07) | 2.65 (2.32-3.15) |  |
| FGF-5 | P12034 | 1 | 1.05 | 96% |  |  |  |  |  |  |  |
| Flt3L | P49771 | 1 | 2.03 | 0% | 8.72 (8.33-8.99) | 8.64 (8.47-8.82) |  |  | 8.98 (8.58-9.14) | 8.65 (8.42-8.87) | 0.005 |
| FS | P19883 | 2 | 1.43 | 1% | 11.64 (11.28-12.20) | 11.73 (11.47-12.05) |  |  | 12.61 (12.44-12.80) | 12.63 (12.34-12.95) |  |
| Gal-9 | O00182 | 2 | 1.84 | 1% | 7.31 (7.18-7.62) | 7.36 (7.25-7.47) |  |  | 7.56 (7.38-7.86) | 7.43 (7.32-7.57) | 0.017 |
| GDF-2 | Q9UK05 | 2 | 2.03 | 1% | 8.97 (8.63-9.36) | 9.00 (8.87-9.29) |  |  | 8.92 (8.80-9.43) | 8.98 (8.69-9.30) |  |
| GDNF | P39905 | 1 | 2.00 | 66% | 1.51 (1.00-2.19) | 1.00 (1.00-2.21) |  |  | 1.00 (1.00-1.00) | 1.00 (1.00-1.00) |  |
| GH | P01241 | 2 | 1.58 | 1% | 10.31 (9.61-10.72) | 10.18 (9.48-10.55) |  |  | 9.00 (8.90-9.29) | 8.95 (8.86-9.13) |  |
| GIF | P27352 | 2 | 1.57 | 1% | 6.02 (5.70-6.60) | 6.28 (5.92-6.69) |  |  | 6.16 (5.64-6.58) | 6.16 (5.82-6.48) |  |
| GLO1 | Q04760 | 2 | 1.62 | 1% | 7.06 (6.49-7.53) | 6.99 (6.77-7.26) |  |  | 7.14 (6.87-7.64) | 6.94 (6.67-7.36) |  |
| GT | P51161 | 2 | 0.76 | 1% | 1.55 (1.33-1.94) | 1.59 (1.46-1.91) |  |  | 1.46 (1.29-1.76) | 1.54 (1.32-1.76) |  |
| HAOX1 | Q9UJM8 | 2 | 1.81 | 1% | 4.77 (4.04-6.28) | 4.19 (3.66-5.08) | 0.035 |  | 6.23 (4.53-6.94) | 5.13 (4.58-5.96) | 0.041 |
| HB-EGF | Q99075 | 2 | 1.98 | 1% | 5.25 (4.96-5.58) | 5.19 (4.96-5.41) |  |  | 5.23 (4.71-5.88) | 5.06 (4.82-5.28) |  |
| HGF | P14210 | 1 | 1.19 | 0% | 8.37 (8.10-8.59) | 8.20 (8.02-8.36) | 0.011 |  | 8.63 (8.38-8.84) | 8.42 (8.15-8.63) | 0.023 |
| HO-1 | P09601 | 2 | 1.19 | 1% | 11.46 (11.21-11.74) | 11.44 (11.22-11.58) |  |  | 11.36 (11.09-11.62) | 11.24 (10.89-11.40) |  |
| hOSCAR | Q8IYS5 | 2 | 2.73 | 1% | 10.53 (10.39-10.61) | 10.52 (10.40-10.66) |  |  | 10.56 (10.41-10.68) | 10.52 (10.45-10.61) |  |
| HSP 27 | P04792 | 2 | 2.52 | 1% | 9.82 (9.49-10.10) | 9.91 (9.66-10.04) |  |  | 9.94 (9.87-10.07) | 9.95 (9.65-10.16) |  |
| IDUA | P35475 | 2 | -0.17 | 1% | 5.44 (5.16-5.76) | 5.59 (5.20-5.90) |  |  | 5.56 (5.37-5.77) | 5.61 (5.32-5.89) |  |
| IFN-gamma | P01579 | 1 | 3.79 | 0% | 5.69 (5.16-6.30) | 5.64 (5.15-6.13) |  |  | 5.75 (5.29-6.43) | 5.51 (5.14-6.02) |  |
| IgG Fc receptor II-b | P31994 | 2 | 2.26 | 2% | 3.85 (3.62-4.00) | 3.75 (3.20-4.15) |  |  | 3.74 (3.53-4.02) | 3.76 (3.18-4.18) |  |
| IL-1 alpha | P01583 | 1 | 0.45 | 96% |  |  |  |  |  |  |  |
| IL-10 | P22301 | 1 | 1.93 | 0% | 3.60 (3.26-3.90) | 3.50 (3.20-3.67) |  |  | 3.59 (3.45-3.93) | 3.42 (3.17-3.57) | 0.001 |
| IL-10RA | Q13651 | 1 | 0.76 | 45% | 0.79 (-0.24-0.99) | 0.80 (-0.24-1.11) |  |  | 0.81 (-0.24-0.97) | 0.80 (-0.24-1.09) |  |
| IL-10RB | Q08334 | 1 | 1.27 | 0% | 5.47 (5.31-5.66) | 5.44 (5.35-5.62) |  |  | 5.85 (5.75-6.00) | 5.75 (5.58-5.95) | 0.035 |
| IL-12B | P29460 | 1 | 0.54 | 0% | 5.46 (5.15-5.91) | 5.48 (5.22-5.85) |  |  | 5.05 (4.69-5.62) | 5.10 (4.77-5.37) |  |
| IL-13 | P35225 | 1 | 1.37 | 89% |  |  |  |  |  |  |  |
| IL-15RA | Q13261 | 1 | 0.97 | 33% | 1.06 (-0.03-1.18) | 0.98 (-0.03-1.13) |  |  | 1.17 (1.02-1.26) | 1.05 (0.97-1.24) |  |
| IL-16 | Q14005 | 2 | 1.00 | 1% | 5.93 (5.76-6.21) | 5.98 (5.67-6.23) |  |  | 5.75 (5.51-6.06) | 5.73 (5.47-5.95) |  |
| IL-17A | Q16552 | 1 | 1.30 | 64% | 0.30 (0.30-1.56) | 0.30 (0.30-1.46) |  |  | 0.30 (0.30-1.36) | 0.30 (0.30-0.30) |  |
| IL-17C | Q9P0M4 | 1 | 1.62 | 86% |  |  |  |  |  |  |  |
| IL-17D | Q8TAD2 | 2 | 2.14 | 17% | 2.52 (2.36-2.68) | 2.50 (2.32-2.62) |  |  | 2.27 (1.14-2.41) | 2.21 (1.08-2.38) |  |
| IL-18 | Q14116 | 1 | 0.62 | 0% | 7.84 (7.42-8.04) | 7.50 (7.29-7.92) |  |  | 9.06 (8.53-9.44) | 8.66 (8.48-8.92) | 0.009 |
| IL-18 | Q14116 | 2 | 1.92 | 1% | 8.49 (8.07-8.81) | 8.19 (7.99-8.64) |  |  | 9.06 (8.53-9.44) | 8.66 (8.48-8.92) | 0.019 |
| IL-18R1 | Q13478 | 1 | 1.75 | 0% | 7.89 (7.60-8.11) | 7.91 (7.54-8.08) |  |  | 8.25 (7.63-8.47) | 7.89 (7.69-8.16) | 0.047 |
| IL-1ra | P18510 | 2 | 1.14 | 1% | 4.70 (4.42-5.22) | 4.58 (4.30-4.87) |  |  | 5.18 (4.85-6.22) | 4.79 (4.63-5.00) | <0.001 |
| IL1RL2 | Q9HB29 | 2 | 1.80 | 1% | 4.34 (4.02-4.61) | 4.45 (4.25-4.66) |  |  | 3.73 (3.46-4.01) | 3.68 (3.44-3.88) |  |
| IL-2 | P60568 | 1 | 1.67 | 100% |  |  |  |  |  |  |  |
| IL-20 | Q9NYY1 | 1 | 1.04 | 96% |  |  |  |  |  |  |  |
| IL-20RA | Q9UHF4 | 1 | 1.31 | 88% |  |  |  |  |  |  |  |
| IL-22RA1 | Q8N6P7 | 1 | 2.48 | 94% |  |  |  |  |  |  |  |
| IL-24 | Q13007 | 1 | 2.13 | 94% |  |  |  |  |  |  |  |
| IL-27 | Q8NEV9,Q14213 | 2 | 1.40 | 1% | 7.84 (7.44-8.12) | 7.81 (7.54-8.04) |  |  | 8.02 (7.92-8.08) | 8.04 (7.96-8.13) |  |
| IL-2RB | P14784 | 1 | 1.77 | 95% |  |  |  |  |  |  |  |
| IL-33 | O95760 | 1 | 1.60 | 98% |  |  |  |  |  |  |  |
| IL-4 | P05112 | 1 | 1.04 | 86% |  |  |  |  |  |  |  |
| IL-4RA | P24394 | 2 | 1.20 | 1% | 2.41 (2.28-2.58) | 2.41 (2.20-2.55) |  |  | 2.96 (2.67-3.15) | 2.76 (2.59-2.96) |  |
| IL-5 | P05113 | 1 | 1.35 | 80% |  |  |  |  |  |  |  |
| IL-6 | P05231 | 1 | 1.76 | 2% | 2.50 (2.15-2.82) | 2.42 (2.10-2.68) |  |  | 2.80 (2.47-3.32) | 2.37 (2.16-2.66) | <0.001 |
| IL-6 | P05231 | 2 | 1.90 | 1% | 2.81 (2.51-3.17) | 2.67 (2.37-2.96) |  |  | 3.14 (2.82-3.64) | 2.71 (2.48-2.90) | <0.001 |
| IL-7 | P13232 | 1 | 0.83 | 0% | 2.47 (2.30-3.25) | 2.44 (2.12-2.82) |  |  | 2.79 (2.16-3.29) | 2.38 (2.04-2.68) |  |
| ITGB1BP2 | Q9UKP3 | 2 | 3.25 | 50% | 2.77 (2.04-3.92) | 2.21 (1.96-3.45) |  |  | 3.37 (1.96-3.86) | 2.18 (1.99-3.65) |  |
| KIM-1 | Q96D42 | 2 | 2.61 | 1% | 7.90 (7.45-8.36) | 7.83 (7.66-8.19) |  |  | 8.61 (8.24-9.08) | 8.32 (8.08-8.68) |  |
| KITLG | P21583 | 1 | 1.20 | 0% | 8.64 (8.38-8.83) | 8.55 (8.33-8.80) |  |  | 8.32 (8.20-8.58) | 8.21 (8.08-8.42) |  |
| KITLG | P21583 | 2 | 1.88 | 1% | 9.19 (8.93-9.30) | 9.09 (8.87-9.25) |  |  | 8.97 (8.73-9.15) | 8.80 (8.69-9.02) |  |
| LAP TGF-beta-1 | P01137 | 1 | 1.11 | 0% | 7.09 (6.68-7.28) | 6.89 (6.77-7.15) |  |  | 7.76 (7.41-7.96) | 7.50 (7.24-7.76) | 0.013 |
| LEP | P41159 | 2 | 1.91 | 1% | 6.94 (6.56-7.39) | 6.77 (6.34-7.14) |  |  | 7.27 (6.63-7.66) | 6.98 (6.47-7.27) |  |
| LIF | P15018 | 1 | 0.84 | 91% |  |  |  |  |  |  |  |
| LIF-R | P42702 | 1 | 1.41 | 0% | 4.54 (4.18-4.82) | 4.56 (4.24-4.80) |  |  | 5.92 (5.45-6.17) | 5.78 (5.29-6.05) |  |
| LOX-1 | P78380 | 2 | 1.66 | 1% | 7.57 (7.18-7.73) | 7.24 (6.97-7.53) | 0.035 |  | 8.69 (8.18-8.95) | 8.27 (7.91-8.51) | 0.003 |
| LPL | P06858 | 2 | 2.84 | 1% | 10.37 (10.16-10.48) | 10.37 (10.20-10.48) |  |  | 9.80 (9.60-9.99) | 9.76 (9.48-9.99) |  |
| LT-alpha (TNF-beta) | P01374 | 1 | 1.42 | 0% | 4.37 (4.14-4.61) | 4.49 (4.23-4.64) |  |  | 4.32 (4.14-4.47) | 4.34 (4.08-4.56) |  |
| MARCO | Q9UEW3 | 2 | 2.36 | 1% | 6.90 (6.78-7.02) | 6.89 (6.75-7.05) |  |  | 7.02 (6.85-7.10) | 6.94 (6.74-7.12) |  |
| M-CSF (CSF-1) | P09603 | 1 | 0.91 | 0% | 9.84 (9.70-10.10) | 9.74 (9.63-9.91) | 0.019 |  | 10.14 (10.01-10.26) | 9.95 (9.86-10.07) | <0.001 |
| MERTK | Q12866 | 2 | 2.43 | 1% | 6.41 (6.19-6.65) | 6.42 (6.19-6.61) |  |  | 6.67 (6.34-6.94) | 6.56 (6.35-6.73) |  |
| MMP-1 | P03956 | 1 | 1.91 | 0% | 8.59 (7.74-9.24) | 8.19 (7.54-8.87) |  |  | 9.24 (8.23-9.98) | 8.43 (7.69-9.06) | 0.009 |
| MMP-10 | P09238 | 1 | 1.30 | 0% | 7.34 (7.01-7.74) | 7.48 (7.27-7.86) |  |  | 7.19 (6.93-7.47) | 7.27 (6.99-7.51) |  |
| MMP-12 | P39900 | 2 | 1.98 | 1% | 8.56 (8.05-8.91) | 8.81 (8.24-9.22) |  |  | 7.82 (7.30-8.04) | 7.81 (7.39-8.22) |  |
| MMP-7 | P09237 | 2 | 1.33 | 1% | 9.63 (9.40-9.85) | 9.70 (9.56-9.84) |  |  | 9.77 (9.59-10.11) | 9.78 (9.53-10.05) |  |
| NEMO | Q9Y6K9 | 2 | 1.83 | 1% | 4.85 (4.31-5.66) | 4.68 (4.45-5.06) |  |  | 4.85 (4.52-5.34) | 4.77 (4.28-5.22) |  |
| NRTN | Q99748 | 1 | 1.07 | 88% |  |  |  |  |  |  |  |
| NT-3 | P20783 | 1 | 1.66 | 14% | 2.15 (1.88-2.43) | 2.07 (1.82-2.32) |  |  | 1.85 (0.66-1.94) | 1.96 (1.73-2.21) |  |
| OPG | O00300 | 1 | 1.24 | 0% | 10.11 (9.86-10.56) | 10.00 (9.81-10.28) |  |  | 10.79 (10.40-11.18) | 10.38 (10.13-10.88) | 0.016 |
| OSM | P13725 | 1 | 0.41 | 0% | 4.88 (4.18-5.68) | 4.46 (3.88-5.04) | 0.037 |  | 5.37 (4.25-5.73) | 5.03 (4.47-5.36) |  |
| PAPP-A | Q13219 | 2 | 2.20 | 1% | 9.57 (9.03-9.93) | 9.69 (9.15-9.95) |  |  | 9.49 (9.06-9.72) | 9.54 (9.17-9.77) |  |
| PAR-1 | P25116 | 2 | 1.71 | 1% | 8.09 (7.99-8.32) | 8.15 (7.92-8.29) |  |  | 8.22 (8.04-8.41) | 8.09 (7.93-8.32) |  |
| PARP-1 | P09874 | 2 | 2.11 | 1% | 3.32 (2.98-3.49) | 3.29 (3.13-3.53) |  |  | 3.45 (3.24-3.67) | 3.17 (2.91-3.42) | 0.002 |
| PDGF subunit B | P01127 | 2 | 1.50 | 1% | 9.03 (8.39-9.55) | 8.73 (8.21-9.36) |  |  | 8.76 (8.26-9.91) | 8.72 (7.95-9.34) |  |
| PD-L1 | Q9NZQ7 | 1 | 2.41 | 0% | 6.45 (6.16-6.77) | 6.48 (6.27-6.66) |  |  | 8.12 (7.92-8.26) | 7.99 (7.56-8.32) |  |
| PD-L2 | Q9BQ51 | 2 | 1.64 | 1% | 3.65 (3.44-3.75) | 3.67 (3.53-3.78) |  |  | 4.04 (3.63-4.23) | 4.05 (3.95-4.30) |  |
| PIgR | P01833 | 2 | 3.17 | 1% | 5.56 (5.49-5.64) | 5.53 (5.48-5.59) |  |  | 5.58 (5.46-5.65) | 5.56 (5.48-5.62) |  |
| PLGF | P49763 | 2 | 1.13 | 1% | 7.93 (7.77-8.07) | 7.93 (7.69-8.16) |  |  | 10.95 (10.37-11.58) | 11.26 (10.72-11.85) |  |
| PRELP | P51888 | 2 | 2.17 | 1% | 7.90 (7.82-8.01) | 7.89 (7.82-7.99) |  |  | 7.83 (7.73-7.93) | 7.81 (7.71-7.86) |  |
| PRSS27 | Q9BQR3 | 2 | 1.10 | 1% | 8.68 (8.28-8.87) | 8.62 (8.31-8.96) |  |  | 8.93 (8.62-9.31) | 8.83 (8.52-9.09) |  |
| PRSS8 | Q16651 | 2 | 0.25 | 1% | 8.75 (8.54-9.03) | 8.82 (8.65-9.00) |  |  | 9.55 (9.36-9.59) | 9.49 (9.40-9.56) |  |
| PSGL-1 | Q14242 | 2 | 1.35 | 1% | 4.22 (4.09-4.31) | 4.21 (4.10-4.33) |  |  | 4.26 (4.11-4.32) | 4.14 (4.02-4.31) |  |
| PTX3 | P26022 | 2 | 1.52 | 1% | 3.14 (2.91-3.37) | 3.21 (3.00-3.35) |  |  | 3.27 (3.10-3.45) | 3.17 (3.03-3.31) |  |
| RAGE | Q15109 | 2 | 1.28 | 1% | 13.05 (12.88-13.17) | 13.15 (12.99-13.35) | 0.016 |  | 12.88 (12.63-13.13) | 12.98 (12.78-13.20) |  |
| RANK (TNFRSF11A) | Q9Y6Q6 | 2 | 0.92 | 1% | 5.36 (5.11-5.56) | 5.32 (5.10-5.52) |  |  | 5.87 (5.60-6.14) | 5.52 (5.43-5.70) | 0.001 |
| REN | P00797 | 2 | 1.73 | 1% | 7.91 (7.72-8.20) | 8.10 (7.92-8.31) | 0.015 |  | 7.78 (7.60-8.08) | 7.70 (7.51-7.92) |  |
| S100A12 | P80511 | 1 | 0.41 | 0% | 2.09 (1.76-2.38) | 1.97 (1.75-2.25) |  |  | 1.61 (1.24-2.02) | 1.29 (1.05-1.56) | 0.010 |
| SERPINA12 | Q8IW75 | 2 | 1.53 | 1% | 4.56 (3.94-5.27) | 4.17 (3.75-5.04) |  |  | 5.11 (4.61-6.47) | 4.85 (4.43-5.69) |  |
| SIRT2 | Q8IXJ6 | 1 | 2.45 | 12% | 3.19 (2.81-4.13) | 3.07 (2.67-3.58) |  |  | 3.28 (2.87-3.99) | 3.15 (2.63-3.54) |  |
| SLAMF1 | Q13291 | 1 | 1.68 | 98% |  |  |  |  |  |  |  |
| SLAMF7 | Q9NQ25 | 2 | 2.25 | 11% | 2.82 (2.56-3.20) | 2.84 (2.64-3.17) |  |  | 2.76 (2.40-3.11) | 2.65 (2.41-2.87) |  |
| SOD2 | P04179 | 2 | 1.50 | 1% | 9.97 (9.89-10.03) | 10.00 (9.94-10.03) |  |  | 9.83 (9.77-9.95) | 9.86 (9.79-9.91) |  |
| SORT1 | Q99523 | 2 | 1.60 | 1% | 7.71 (7.56-7.93) | 7.69 (7.60-7.83) |  |  | 7.99 (7.85-8.05) | 7.94 (7.75-8.05) |  |
| SPON2 | Q9BUD6 | 2 | 0.63 | 1% | 8.25 (8.16-8.34) | 8.28 (8.21-8.34) |  |  | 8.36 (8.28-8.40) | 8.29 (8.21-8.36) | 0.041 |
| SRC | P12931 | 2 | 1.28 | 1% | 6.18 (5.53-6.86) | 6.04 (5.65-6.53) |  |  | 6.00 (5.75-6.80) | 6.09 (5.36-6.51) |  |
| ST1A1 | P50225 | 1 | 1.78 | 59% | 0.78 (0.78-1.88) | 0.78 (0.78-2.01) |  |  | 1.85 (0.78-2.20) | 0.78 (0.78-2.04) |  |
| STAMBP | O95630 | 1 | 0.90 | 0% | 4.10 (3.91-4.89) | 4.11 (3.84-4.49) |  |  | 4.22 (3.79-4.76) | 4.02 (3.74-4.44) |  |
| STK4 | Q13043 | 2 | 1.24 | 2% | 3.78 (3.21-4.61) | 3.66 (3.19-4.01) |  |  | 3.68 (3.45-4.10) | 3.61 (2.78-4.00) |  |
| TF | P13726 | 2 | 1.68 | 1% | 5.49 (5.33-5.68) | 5.46 (5.36-5.61) |  |  | 5.37 (5.22-5.56) | 5.34 (5.24-5.48) |  |
| TGF-alpha | P01135 | 1 | 0.41 | 0% | 2.82 (2.63-3.08) | 2.71 (2.64-2.91) |  |  | 2.93 (2.80-3.09) | 2.85 (2.73-2.98) |  |
| TGM2 | P21980 | 2 | 2.70 | 1% | 7.37 (6.89-7.96) | 7.27 (6.78-7.80) |  |  | 7.53 (7.18-7.88) | 7.54 (7.19-7.93) |  |
| THBS2 | P35442 | 2 | 0.52 | 1% | 5.44 (5.32-5.50) | 5.41 (5.35-5.49) |  |  | 5.45 (5.39-5.53) | 5.40 (5.32-5.52) |  |
| THPO | P40225 | 2 | 1.12 | 1% | 3.49 (3.34-3.66) | 3.52 (3.37-3.62) |  |  | 3.59 (3.45-3.76) | 3.50 (3.38-3.67) |  |
| TIE2 | Q02763 | 2 | 1.57 | 1% | 7.21 (7.10-7.40) | 7.22 (7.11-7.37) |  |  | 7.15 (7.01-7.31) | 7.20 (7.07-7.33) |  |
| TM | P07204 | 2 | 3.09 | 1% | 9.37 (9.12-9.51) | 9.33 (9.18-9.49) |  |  | 9.52 (9.40-9.61) | 9.42 (9.31-9.55) |  |
| TNF | P01375 | 1 | 0.09 | 0% | 2.35 (2.17-2.64) | 2.32 (2.04-2.61) |  |  | 2.53 (2.20-2.77) | 2.32 (2.14-2.56) |  |
| TNFRSF13B | O14836 | 2 | 1.91 | 1% | 9.53 (9.37-9.84) | 9.60 (9.46-9.87) |  |  | 9.47 (9.33-9.80) | 9.62 (9.35-9.77) |  |
| TNFRSF9 | Q07011 | 1 | 2.07 | 0% | 6.19 (5.91-6.43) | 6.14 (6.03-6.41) |  |  | 6.01 (5.78-6.21) | 5.95 (5.79-6.14) |  |
| TNFSF14 | O43557 | 1 | 1.59 | 0% | 3.46 (3.17-3.80) | 3.33 (3.07-3.54) | 0.027 |  | 3.27 (3.05-3.55) | 3.16 (2.97-3.26) |  |
| TRAIL | P50591 | 1 | 0.73 | 0% | 7.47 (7.36-7.59) | 7.37 (7.21-7.59) |  |  | 7.44 (7.21-7.62) | 7.26 (7.07-7.46) | 0.025 |
| TRAIL-R1 (TNFRSF10A) | O00220 | 2 | 1.77 | 1% | 2.83 (2.63-2.94) | 2.78 (2.66-2.85) |  |  | 3.11 (2.84-3.31) | 2.94 (2.83-3.11) | 0.033 |
| TRAIL-R2 | O14763 | 2 | 1.87 | 1% | 5.69 (5.55-5.93) | 5.64 (5.58-5.81) |  |  | 5.97 (5.75-6.18) | 5.84 (5.67-5.98) | 0.042 |
| TRANCE | O14788 | 1 | 0.78 | 0% | 3.40 (3.03-3.81) | 3.35 (3.00-3.89) |  |  | 2.89 (2.36-3.26) | 2.84 (2.36-3.18) |  |
| TSLP | Q969D9 | 1 | 0.85 | 96% |  |  |  |  |  |  |  |
| TWEAK | O43508 | 1 | 0.72 | 0% | 8.99 (8.84-9.18) | 8.93 (8.82-9.16) |  |  | 8.43 (8.16-8.66) | 8.50 (8.29-8.74) |  |
| uPA | P00749 | 1 | 1.56 | 0% | 9.87 (9.75-10.00) | 9.82 (9.69-10.02) |  |  | 10.42 (10.07-10.60) | 10.27 (10.09-10.44) |  |
| VEGF-A | P15692 | 1 | 0.60 | 0% | 9.90 (9.79-10.10) | 9.81 (9.70-9.90) | 0.018 |  | 10.20 (10.01-10.31) | 10.05 (9.92-10.14) | 0.002 |
| VEGF-D | O43915 | 2 | 0.84 | 1% | 7.70 (7.44-8.03) | 7.93 (7.63-8.15) |  |  | 7.42 (7.24-7.86) | 7.59 (7.34-7.80) |  |
| VSIG2 | Q96IQ7 | 2 | 1.75 | 1% | 3.21 (3.00-3.34) | 3.18 (3.01-3.40) |  |  | 3.13 (2.94-3.35) | 3.13 (2.92-3.31) |  |
| XCL1 | P47992 | 2 | 0.92 | 1% | 5.05 (4.78-5.21) | 5.07 (4.78-5.39) |  |  | 5.01 (4.71-5.36) | 4.90 (4.63-5.18) |  |

LOD=Limit of Detection; Percentages indicate <LOD for sampling occasions in both the first and second trimesters; Trim1=First trimester; Trim2=Second trimester.

Panel 1=Olink Target 96 Inflammation panel. Panel 2=Olink Target 96 Cardiovascular II panel.
